# Representing subpopulations with latent profile analysis: a non-technical introduction using exercisers’ goal orientation adoption profiles

**DOI:** 10.1007/s10865-025-00596-5

**Published:** 2025-09-09

**Authors:** E. Whitney G. Moore, Alessandro Quartiroli

**Affiliations:** 1 Department of Kinesiology, East Carolina University, Greenville, NC USA; 2 Department of Psychology, University of Wisconsin–La Crosse, La Crosse, WI USA; 3 School of Psychology, Sport, and Health Sciences, University of Portsmouth, Portsmouth, England

**Keywords:** Finite mixture model, Latent profile analysis, Latent class cluster analysis, Goal orientations, Achievement goal theory

## Abstract

Latent profile analysis (LPA) is in the finite mixture model analysis family and identifies subgroups by participants’ responses to continuous variables (i.e., indicators); participants’ probable membership in each subgroup is based on the similarity between the subgroup’s prototypical responses and the person’s unique responses. Compared to latent class analysis (LCA) with categorical data, LPA is a better fit for many variables and theories in behavioral medicine, because LPA can have continuous item, sub-scale, or scale scores as indicators, which can enable identifying and examining subgroups defined by responses representing complex, multidimensional concepts (e.g., orientations, motivations, well-being, ill-being, physical activity engagement) and biomarkers of diseases and disorders. Recently, the use of LPA has increased and as it continues to evolve, it is important researchers know best practice recommendations and explanations for both conducting as well as reading/reviewing LPA models. With this paper we: 1) discuss the strengths and weaknesses of LPA and the questions it is most appropriate to answer, 2) introduce LPA conceptually, 3) illustrate an LPA conducted with exercise psychology variables following current best practice recommendations, and 4) juxtapose resulting models from the LPA approach to a typical approach with the same data. We also share the data and syntax files used to conduct the basic steps of the LPA analyses as supplemental appendix files in addition to including the tables and figures for reporting LPA results following best practices.

## Introduction

Finite mixture models are increasingly favored for person-centered analytical approaches (Collins & Lanza, 2010; Wang & Wang, 2020), complementing traditional variable-centered methods (Moore & Little, 2024; Wang & Wang, 2020). Finite mixture models facilitate the exploration, identification, and description of distinct, mutually exclusive latent subgroups (i.e., profiles) based on individuals' response patterns to specific variables (also called indicators; Collins & Lanza, 2010; Nylund-Gibson & Choi, 2018). Rather than assuming a uniform response pattern across the entire sample, finite mixture models assume there are two or more distinct subgroups exhibiting unique response patterns (Nylund-Gibson & Choi, 2018; Collins & Lanza, 2010). Finite mixture models are characterized as ‘finite’ because a limited number of mutually exclusive and distinct profiles (i.e., latent subgroups or subpopulations) are determined to represent the heterogeneity of indicator responses across participants in the sample (Masyn, 2013; Moore & Little, 2024). The weighted combination of the ‘mixture’ of the profiles’ characteristics (e.g., means, variances, covariances, skew, kurtosis) represents the overall sample characteristics. Thus, the distribution of indicator scores is considered a mixture of the two or more profiles’ distributions. These models assume a mixture of distributions, generally characterized by heterogeneity across the sample and homogeneity within subpopulations (Rosato & Baer, 2012). Assuming two or more profiles exist, finite mixture models can enable researchers to (a) determine the number and structural composition of the profiles that best represents the data characteristics (e.g., means, variances, covariances, skew, kurtosis); (b) examine the unique characteristics of each profile; (c) test the whether covariates significantly predict membership between the profiles (e.g., odds of membership in Profile 1 compared to Profile 2 significantly differs by gender identity); and (d) the ability of profile membership to predict significant different levels on outcome variables. The purpose of this article is to introduce when and how to conduct and interpret a latent profile analysis (LPA), a specific finite mixture model type.

The focus of this article is on LPA models because they are conducted with continuous indicators (Masyn, 2013; Moore & Little, 2024), which are common in behavioral health research. Continuous indicators can be individual item, subscale, or scale scores, as well as physiological measures. For example, Masyn (2013) illustrated the application of LPA using three widely accepted clinical indicators for diagnosing diabetes: glucose intolerance (area under the curve for plasma glucose), insulin response to oral glucose (area under the curve for oral glucose), and insulin resistance (steady state plasma glucose). In her example, diabetes diagnosis status, based on traditional univariate clinical cut-offs, was subsequently used as a predictor of profile membership. While the three identified profiles showed substantial overlap with existing clinical diagnoses; approximately 15% were classified differently, highlighting the potential of LPA to refine diagnostics using multivariate criterion or identify individuals at greater risk than conventional, univariate criterion suggest. Therefore, the application of LPA in behavioral health research offers opportunities to uncover meaningful subpopulations and inform the development of more targeted, impactful interventions.

### Variable-centered and person-centered statistical methods

Understanding the distinctiveness of person-centered methods (e.g., k-means, hierarchical clustering, latent profile/class analysis, growth mixture model) in contrast to traditional variable-centered statistical methods (e.g., ANOVA, regression analysis, factor analysis) is crucial (Wang et al., 2013). Variable-centered methods primarily explore interrelationships among variables and infer underlying processes, assuming a homogeneous population (Wang et al., 2013). Variable-centered approaches handle observable group differences (e.g., age, sport, gender identity). However, they fall short when identifying quantitatively or qualitatively distinct groups (i.e., profiles) and their differential indicator relationships; a task better addressed by person-centered methods like finite mixture models, which explore unobserved (latent) subpopulations (Hofmans et al., 2020; Wang et al., 2013). Unobserved population heterogeneity occurs when the heterogeneity is due to unobservable (i.e., latent) subpopulations or subgroups (i.e., profiles). As these groupings are not due to values on known grouping variables, these sub-populations are referred as latent profiles (Hofmans et al., 2020). Person-centered analytical methods not only focus on the relations between variables but also the relations among individuals (Zyphur, 2009). These methods classify individuals based on the existing similarities in their scores on a set of variables (Howard & Hoffman, 2018; Woo et al., 2018). Thus, rather than assuming population homogeneity, they aim to model unobserved heterogeneity within the population (Woo et al., 2018), identifying unobserved subpopulations comprised of individuals with similar response patterns (Wang & Wang, 2020). Researchers should select appropriate analysis to address their theory-informed research question, while also taking into account the assumptions of the analytical procedure.

Morin et al. (2018) argued that person-centered methods are typological, prototypical, and exploratory. They are typological in the sense that they categorize individuals into qualitatively and quantitatively distinct subpopulations which differ in their sets of variable parameter estimates. The profiles have prototypical response patterns to the indicators defining the profiles. The closer a participant’s responses align with the prototypical response patterns of a profile, the higher the probability that participant is a member of that particular profile. Finally, person-centered analyses are exploratory as the ‘final’ model is typically obtained by comparing the ability of different model solutions with different numbers of profiles to best represent the heterogeneity of the population. The need to compare multiple model solutions is also because there are not objective fit tests for the model fit to the data, which is why even when a more confirmatory approach is taken with mixture models, multiple different profile model solutions need to be estimated to determine the best fitting model (Masyn, 2013; Van Lissa et al., 2024). While there are multiple finite mixture model types (see Moore & Little, 2024; Petras & Masyn, 2013 for more information), in the following sections, we introduce LPA contextually and then present the steps to implement it along with the results from an exercise psychology data example.

### Latent profile analysis (LPA)

LPA (sometimes called also Latent Class Cluster Analysis—LCCA) is useful when patterns of shared behaviors or characteristics between and within subgroups may be missed when conducting variable-centered analyses (Ferguson et al., 2020; Moore & Little, 2024). LPA focuses on identifying latent profiles within a population based on the individuals’ patterns of responses to specific continuous indicators (Lanza et al., 2013; Howard & Hoffman, 2018; Wang & Wang, 2020). Indicators are assumed to be independent of each other (i.e., assumption of local or conditional independence), thus their observed interrelatedness is considered caused by the mixture of subpopulations while the within-profile observed interrelatedness is considered as measurement error or residuals (Spurk et al., 2020). This assumption is sometimes relaxed allowing indicators to correlate with each other within a profile to be used to distinguish profiles (Nylund-Gibson & Choi, 2018). The indicators’ variances may also be constrained to equality across profiles or freely estimated (Masyn, 2013).

The latent profile construct is an unobserved, nominal categorical latent construct representing the finite number of profiles enumerated in the model. LPA assumes individuals in the same sample can be typed with varying degrees of probabilities into different profiles based on their pattern of responses to specific indicators. The better an individual’s response pattern matches the prototypical response pattern of a particular profile, the higher their probability of being a member of that profile. The profile in which a person has the highest probability of membership is called their modal profile. While modal profile membership is mutually exclusive (i.e., participants can only be a member of one profile in a model), an important and unique aspect of LPA is that the uncertainty of assignment to a given profile for an individual is represented in the LPA model results by the membership probabilities. When covariates are added into the model (see Covariate Section), this uncertainty continues to be modeled in LPA.

#### Data example

The exercise psychology data example we will use to concretely illustrate the steps of the LPA is comprised of the task and ego goal orientations (strongly disagree *(1)* to strongly agree *(7)*) reported by 697 participants in 47 college group fitness classes (e.g., yoga, weightlifting, kickboxing, basketball) at the middle of the semester for the profiles (Fig. 1A).^1^ The participants’ reported gender identity (40% men; 60% women) is used as a predictor of profile membership. The students’ PE enjoyment and boredom at the end of the semester were tested for significant differences as outcomes of profile membership (Fig. 1B). According to Nicholls’ Achievement Goal Perspective Theory, task and ego goal orientations are orthogonal; thus, individuals’ adoption of one goal orientation does not influence their adoption of the other goal orientation. In variable-centered analyses, task and ego orientations are modeled as two separate variables, with low to no correlation between them, and relationships with other variables are interpreted separately for task goal orientation and ego goal orientation. This does not represent the reality of how an individual adopting both task and ego goal orientations highly may have a different enjoyment level than someone who moderately adopts a task orientation and highly adopts an ego orientation. This real-world experience, however, can be represented with an LPA model using the goal orientations as the indicators of a latent profile construct. Thus, this is an example where the analytical techniques have advanced to facilitate testing the theory as it was originally conceptualized by Nicholls (1989).

Participants’ gender identity was included as a predictor of the goal orientation profiles, because researchers have shown gender differences. Specifically, in a meta-analysis, women reported higher task orientation compared to men, while men reported higher ego orientation levels than women (Lochbaum et al., 2016a, 2016b). In our data, compared to women, men reported significantly higher ego orientations and lower task orientations. Furthermore, task goal orientation has been positively correlated with positive emotions (e.g., enjoyment) and negatively with negative emotions (e.g., boredom), while ego orientation tends to have low to nonsignificant relationships with positive and negative emotions. This lack of relationship may represent a cancelation effect of different correlations existing with different ego orientation levels (Lochbaum et al., 2016a, 2016b). The results of the variable-centered analysis with the example data aligned with the established patterns presented above: a nonsignificant correlation between the task and ego goal orientations, significant predictive relationships from task orientation to the outcomes (i.e., boredom and enjoyment), and nonsignificant predictive relationships from ego orientation to the outcomes.

The data were collected from participants in group fitness classes at a college; thus, the participant observations were not all independent but nested within classes. There were two options for analyzing this data: LPA accounting for nested structure for parameter estimates or a multilevel LPA. Given, the research question was about individuals’ goal orientations, responses, and gender identity, and not also group effects, we conducted an LPA with the parameter estimates adjusted for the nested nature of the data using the TYPE = COMPLEX command in combination with the TYPE = MIXTURE command in M*plus* 8.9 (Muthen & Muthen, 1998–2017). With this manuscript we focus on introducing LPA modeling steps with an appropriate data example. Those interested in multilevel finite mixture models are referred to Asparouhov and Muthen (2008), Chapter 10 of M*plus* User’s Guide (Muthen & Muthen, 1998–2017), Morgan and Padgett (2021), and Burns et al. (2022).

#### Latent profile analysis research questions and hypotheses

LPA research questions and hypotheses can be a trip point for those new to this analysis type. Rather than hypothesizing a specific number of profiles, we conduct the LPA to determine the profile number and typology based on our selected indicators (Masyn, 2013). We do not have hypotheses regarding the number or composition of the profiles, because this is our measurement structure. Furthermore, because of the nature of finite mixture models, different profile solutions are not nested, so we cannot do nested model, chi-square difference tests (Moore & Little, 2024). We can, however, have research questions about the number of profiles. For the example analysis, the research question could be: How many profiles are needed to best represent college fitness class participants’ goal orientation taxonomy based on their self-reported task and ego orientation levels.

We can have hypotheses about the latent relationships between profile membership and predictors, outcomes, or as a moderator of relationships between other model variables. As the profile construct is a nominal variable, predictors of membership are predicting differences in the odds or likelihood of membership in one profile relative to another profile by their predictor value. However, as we do not know a priori the number or composition of our profiles, these hypotheses need to be general. With our example analysis, the hypothesis with the gender identity predictor is that the probability or odds of profile membership will significantly differ between men and women.

The other common hypothesis type regards outcomes. Although represented in models as a regression line from the latent profile construct to the outcome(s), the test is for mean differences by profile membership (analogous to an ANOVA or ANCOVA analysis if predictors are also present). Again, without knowing the number and composition of the profiles a priori, the outcome hypotheses are also general. With our example analysis, we hypothesize the fitness class participants’ enjoyment and boredom will significantly differ by their profile membership.

##### Power, sample, and profile sizes

It is generally recommended profile sizes are at least 5–8% of the sample (Nylund-Gibson & Choi, 2018). Although with smaller samples this threshold may need to be increased (Masyn, 2013); whereas with larger samples this threshold may be lower. The sample size and power of finite mixture models also depend on the number of parameters estimated, which depend on the number of indicators included, profiles enumerated, variance–covariance structure (i.e., parameters estimated) as well as the quality of the class separation (Masyn, 2013; Tein et al., 2013). The diversity of these unknowns and exploratory nature of finite mixture models, makes it not feasible to conduct power analyses to inform sample sizes for person-centered analyses. While a variety of recommendations in terms of appropriate sample size exist (Finch & Bronk, 2011; Morgan, 2015; Nylund et al., 2007), there is no clear consensus yet. Tein and colleagues (2013) found samples of 300–500 were often used for finite mixture models; however, it is also important to think about the minimum sample size for an interpretable profile. It is recommended that latent profile sample sizes be of a size that is acceptable for conducting known group analyses (e.g., biological sex, diagnosed/undiagnosed grouping). For example, the recommendations for acceptable known sample sizes vary from 30 to 60 per group (Moore & Little, 2024; Vincent & Weir, 2012). With smaller total sample sizes, a limitation for LPA is there may be interesting solutions with profiles too small to comfortably interpret and so are not carried forward; with a larger overall sample size those same profiles may be interpretable.

## How to complete a latent profile analysis

In general terms LPA aims to uncover latent profiles (*k*) of individuals (*i*) characterized by a similar pattern of responses on specific indicators (*j*) that are meaningful and interpretable (Ferguson et al., 2020; Marsh et al., 2009; Masyn, 2013; Sterba, 2013). LPA is generally described as a two-phase analysis (Spurk et al., 2020). In the first phase researchers engage in an iterative modeling process aimed to identify the number of profiles to retain. In the second phase, they explore how covariates relate to profile membership (Masyn, 2013; Sterba, 2013). Ferguson and colleagues (2020) offered a stepwise approach for applied researchers to complete an LPA, starting with the inspection of the data, shifting into the iterative evaluation of the models and exploration of model fit, investigating the profiles, and finally, analyzing the covariates.

### Data inspection and missing data

To begin an LPA, it is important to first explore and clean the data for analysis and checked for standard statistical assumptions (cf. Osborne, 2012; Field, 2018) and missing data (see Muthén & Muthén, 2017; Enders, 2022). LPA is able to handle missing data in the indicators utilizing full-information maximum likelihood (FIML). FIML assumes missing values are at least missing at random (MAR) and has been shown to produce unbiased parameter estimates and standard errors under MAR and missing completely at random (MCAR) conditions (Enders & Bandalos, 2001; Enders, 2022). FIML works by estimating a likelihood function for each individual based on the variables that are present so that all the available data are used (see Enders, 2010). FIML estimators are necessary for estimating finite mixture models, because the latent profile membership by definition is MAR. By default, M*plus* uses the MLR (maximum likelihood robust) estimator with finite mixture models providing more robust standard errors when there are deviations from normality and handling all missing data. It is also important to note that finite mixture models assume normal distributions within each profile, not for the population. Thus, the overall population distribution may be nonnormally distributed, but that does not automatically mean the profile distribution will be. However, software programs may be able to also handle special distributions, such as Poisson for count data when needed.^2^ Rather than assuming these adjustments used in variable-centered analyses are needed, it is recommended to first try the finite mixture models to see if the mixture process itself accounts for the inherent nonnormality of the data.

### Analysis Step 1: iterative evaluation of models

The first analysis step to complete an LPA involves evaluating a series of hypothetically plausible iterative models. This exploratory process is analogous to running an exploratory factor analysis (EFA) to examine the fit and quality with iteratively more factors with the same number of items. EFA reduces the data for simpler interpretation by reducing many items into a smaller number of factors. Finite mixture models reduces the many participants into a smaller number of interpretable profiles. The researcher will start with a model encompassing only one profile and adding one profile at a time, until multiple consecutive *k* + 1 models either fail to converge or converge on an untrustworthy model (e.g., Heywood cases, parameter estimates out of bounds, smallest profile sample size uninterpretable; Masyn, 2013; Moore & Little, 2024).

Similar to other finite mixture models (see Wang & Wang, 2020), LPA models may have a hard time converging. With variable-centered analyses there is a single, best fitting model solution (i.e., model parameters such as means, variances, and covariances) which converges at the *log likelihood (LL)* global maximum. Thus, when a variable-centered analysis converges statistics can be calculated with clear interpretation criterion for how well the model specified by the researcher represents the observed data. The nature of finite mixture models is to estimate specified number of profiles (*k*-profiles) of previously unknown proportions that when combined (i.e., weighted sum) not only represent the observed data means, variances, and covariances, but also the distributional properties of the overall dataset, which can have multiple possible model solutions for each *k*-profile model. As a result, evidence needs to be built supporting the model solution is the best fitting model at the *LL* global maximum rather than any of the other possible solutions at *LL* local maxima. The local maxima simply indicates the estimation process has identified a solution, not the best possible solution. To help build evidence for a model solution being at the global maximum, the model is run multiple times using progressively more random start values (more in next paragraph), so the model is estimated multiple times within each attempt. Thus, we, as researchers, have information about the overall convergence rate of the model and frequency of converging on the apparent global maximum solution *within* each attempt, as well as replication of converging on the same global maximum solution *across* progressive attempts. This information is presented by the software output including the *LL* of all different converged models within an attempt. The best-fitting solution is considered the one with the *LL* value closets to zero (Berlin et al., 2014; Vermunt & Magidson, 2002) and repeated as many times as possible (within and across attempts), indicating a better chance that the model converged on the global maximum (Berlin et al., 2014).

To improve the chances of proper convergence, it is important to improve the likelihood of model convergence at the global maximum. It is possible to improve convergence rates by providing the estimation process with multiple (from 40 to 10,000) randomly generated start values. Random start values are random numbers generated by the software to kickstart the FIML estimation process; each model is estimated for a different randomly generated start value. Therefore, the number of random starts specified by the researcher for each attempt represents the number of models that the software will run for that attempt. Thus, with 40 random starts, there will be 40 models uniquely estimated within that model attempt. When models started with different random starts reach convergence at the same *LL* value it is more likely that the model solution is the global maximum solution.

For each of the models, it is important to record the model *LL*, the number of converged models, the proportion of converged models with the same, lowest *LL*, and the proportion for the smallest profile from the model solution (Moore & Little, 2024). After this first *k*-profile model converges, the parameter estimates from the model solution can be used subsequent parameter start values. This allows the current best estimates for the parameters to be the starting point for the FIML estimation process with the subsequent model run. It is useful to run the model while maintaining the same number of random starts (i.e., number of models estimated per attempt). If the lowest *LL* value is repeated, then it is recommended to run the model again with twice as many starts. If a new solution is reached, then repeating the above steps to re-run the model again with start values from the newest model solution is appropriate. The lowest *LL* value should be replicated within a model attempt and should be repeated in the last two model runs (Spurk et al., 2020); the last model run is recommended to be conducted with the maximum number of random starts supported by the software.^3^ Finally, all the processes can be replicated for the next *k* + 1 model until at least two consecutive *k* + 1 models do not converge on a trustworthy solution (e.g., classes collapse, not interpretable/generalizable smallest class).

While LPAs assume conditional independence among the indicators considering their observed interrelatedness as accounted for by the latent profile (Spurk et al., 2020), this assumption is sometimes relaxed allowing continuous indicators to correlate with each other within a profile (Masyn, 2013; Nylund-Gibson & Choi, 2018). Moreover, their variances may also be constrained to equality across profiles or freely estimated (Masyn, 2013). These possibilities bring to light four general variance–covariance structures. As the variance–covariance structure becomes less restrictive, the number of estimated parameters increases, which can hinder model convergence. The four structures can greatly change the definition of the enumerated profiles and the number of profiles considered to best represent the heterogeneity in the data (Masyn, 2013). Thus, it is recommended all four variance–covariance structures of the model are taken through the above process to enumerate (determine) the best measurement structure for the profiles.

These structures differ in terms of their level of restriction. The most restrictive model is the *profile-invariant, diagonal structure*, where covariances between indicators are constrained to zero and their variances are kept equal (i.e., constrained) across profiles. This first structure is the same as the structure used for k-means cluster analysis. The *profile-varying, diagonal structure* freely estimates the indicators’ variances across profiles, but the covariances continue to be constrained to zero. For these two structures, the latent profile construct represents all the covariation (i.e., covariance) between the indicators. The variability of those indicators, however, is part of the measurement structure for the second structure. For the next two structures, at least part of the covariance between the indicators is included in the measurement structure. In the *profile-invariant, non-diagonal structure* the indicator variances and covariances of the indicators are estimated (i.e., not fixed to zero) but constrained to equality across profiles. Finally, the *profile-varying, non-diagonal structure* freely estimates the variances and covariances of the indicators across the profiles (Masyn, 2013; Moore & Little, 2024); this is the most complex measurement structure as the unique variance and covariance estimates are also part of how the profiles are defined. Thus, differences in variances and covariances can be part of how profiles are assessed for homogeneity and separation. In other words, profiles can be distinguished by the mean values of the indicators as well as by their variances and covariances (Masyn, 2013). The best or active LPA enumeration process includes all four variance–covariance structures, leading to identifying at least one ‘best’ model in each structure. By completing this systematic approach to examining the variance–covariance structures, the researcher actively engaged in the modeling process may identify a modification to a structure (e.g., relaxing or enforcing a constraint across profiles for one or subset of parameters) best represents the data upon examination of the model results and residuals (i.e., observed—model estimated values). An example of this is presented in the “Example Data Convergence” section below. The researcher will determine the best fitting model across these ‘best’ contender models across structures (Masyn, 2013). The process can be completed utilizing information criteria, relative fit indices, and interpretability (Nylund-Gibson & Choi, 2018).

#### Example data convergence

The online supplemental materials (https://tinyurl.com/ExampleLPA) include the reporting tables, figures, example syntax, and data. In addition to providing the information criteria and relative fit indices, Supplemental Table 1 also includes the sample size for the smallest profile. In the rest of this section, we present the information about the quality of the model estimation across the four variance–covariance structures. With the diagonal, profile-invariant structure, we were able to have models converge with trustworthy parameter estimates for the 1-profile through 10-profile models. However, the smallest profile sample sizes were 20 or below for the 8-profile (n = 20), 9-profile (n = 20), and 10-profile (n = 5) models; which is small for supporting interpretability. Noteworthily, the replication of the best *LL* was 74% within multiple model runs for the 5-profile model, yet dropped to range from 5 to 37% for the subsequent 6-profile to 10-profile models.

For the diagonal, profile-varying structure, many of the profile models had difficulty initially converging on a solution. Specifically, the 3-profile model did not converge on a quality solution until start values from the three largest profiles from the 4-profile solution were used to facilitate the initial model estimation. Starting with the 4-profile model, at least one profile had to have the variance fixed to 0.01 because of the consistency of the participants on the mean values for the task goal orientation. Not fixing this variance would result in non-convergence due to matrices with zero variance, which cannot be inverted for computations by the software (i.e., cannot divide by zero). If it did converge, there were warnings regarding Heywood cases or non-positive definite matrices. The smallest profile sample size for the 6-profile solution was 24 participants, and for the 7-profile solution was 9 participants; furthermore, the replication of the *LL* was 5% to 27% for the 5- to 7-profile solutions. Thus, model solutions for over 7 profiles were not attempted.

The non-diagonal, profile-invariant structure was more successful and stable in the model results than the prior structure. Models for the 2- through 8-profile solutions quickly converged on a besting fitting solution that was then repeated across attempts with more random start values. The replication of the best solution of the 8-profile solution was low (4%). Compared to a range of 30%-81% for the other profile solutions. Prior to the 8-profile model, the smallest profile was 27, whereas the smallest profile for the 8-profile model was 20.

Finally, the non-diagonal, profile-varying structure also ran into convergence issues. Starting with the 4-profile model, convergence difficulties began which resulted in needing to fix variances for the task goal orientation mean. More importantly, the best fitting solution for multiple profile solutions had profiles with sample sizes too small for interpretability (Table 1). While the smallest sample size for the 5-profile solution was 110 and repeated across attempts, the solution *LL* was only replicated 7–9% within attempts.

### Analysis Step 2: model fit and interpretability

A variety of fit indices are used to identify and interpret the best fitting solution (see Supplemental Table 1). Since all indices provide different information, it is important to consider them using a holistic and gestalt approach when assessing the quality of model fit while keeping in mind the interpretability of the model and its consistency with the theory. These indices can be classified into two main groups: (a) information criteria, and (b) relative fit indices (Nylund-Gibson & Choi, 2018).

#### Information criteria

These are approximate fit indices (i.e., *AIC, BIC, SABIC, CAIC,* and *AWE*); lower values indicate superior fit (Nylund-Gibson & Choi, 2018). All of these fit indices are calculated using the *LL,* sample size, and some factor to punish for lack of parsimony (See Table 2 for formulas). Thus, although no software calculates all fit indices in the output, all of them are calculable with the information in the model output (i.e., *LL,* sample size, number of parameters estimated). Nylund-Gibson and Choi (2018) also suggest plotting the values of these indices to visually represent their values and more easily inspect them. Some of these fit indices may not reach a lowest value, however, by graphically representing the values, it is possible to see an inflection point or plateaus which can represent the point of “diminishing returns” in the model fit (e.g., less improvement in model fit relative to the increased number of parameters estimated). Through simulation studies with known mixture model solutions, basic guidelines on the behavior of these fit indices have been identified (Nylund et al., 2007). The AWE is most likely to reach a lowest value or bottom out. However, the lowest value of the AWE tends to occur one or two models before the best fitting solution (e.g., when the lowest AWE value occurs with 4-profile solution, the best mixture model representation is usually the 5- or 6-profile solution). The lowest value for the AIC tends to occur with the model that has one or two more profiles than the best mixture model. The BIC and SABIC both tend to have their lowest values align with the best mixture model. By using all of the values from these fit indices and typical behaviors to inform our interpretation of them for narrowing down the potential model solutions to the best one or two contending models per variance–covariance structure.

##### Example data information criteria

 The AWE clearly bottomed out in the first three variance–covariance structure model solutions. The BIC bottomed out for the 4-profile solution of the diagonal, profile-varying structure. The non-diagonal, profile-invariant structure model information criteria are a good example of the inflection point concept. The relative improvement in fit started to plateau between the 5- and 7-profile solutions. This again narrows the focus to these profile models as the contenders for being the best fitting.

#### Relative fit indices

Among these indices, there are the likelihood-ratio tests (LRTs) which compare the fit between two neighboring class models (*k-*profile solution compared to a *k-*1 profile solution) and are interpreted similar to a chi-square nested model test. A significant *p-*value supports the *k*-profile solution as fitting significantly better than the *k*-1 profile solution; a nonsignificant *p*-value supports the more parsimonious, *k*-1 profile solution, because the increased complexity of the *k-*profile solution fit was a nonsignificant improvement (Nylund et al., 2007). These LRT statistics and *p*-values are calculated by the analysis software, and include the *VLMR-LRT* (Vuong-Lo-Mendell-Rubin-LRT)*, adjusted-LRT,* and *BLRT*. The adjusted-LRT is a correction in the calculation of the VLMR-LRT *p*-value, which should be reported rather than the VLMR-LRT *p*-value. The BLRT (bootstrapped LRT) *p*-value is based on a bootstrapped distribution of LRT statistics. The BLRT approach unfortunately, more often than not, results in supporting the more complex, *k*-profile model; thus, not providing information to help researchers make a decision. Given the interpretation that a nonsignificant LRT supports the less complex model, and a significant LRT supports the more complex model, in Supplemental Table 1, the LRT is reported as *k* + 1 profile vs *k*-profile, so that the result for the 2-profile vs 1-profile solution is in the 1-profile model row; and the significant LRT supports the 2-profile model rather than the 1-profile solution. In the diagonal, profile-invariant portion of the table, the bolded, nonsignificant LRT *p*-value is in the 5-profile row, which is the profile model solution supported by the nonsignificant LRT rather than the more complex, 6-profile model solution.

The relative fit indices group also encompasses the relative improvement (*RI*), Bayes Factor (*BF*), and correct model Probability (*cmP*), which are calculated by the researcher (see Supplemental Table 2 for formulas)*.* These indices compare two or more models’ relative fit to each other (Masyn, 2013; Nagin, 1999), and enable the user, in relation to the models’ *LL* and *BIC* values, to determine “*how much*” better one model represents the data compared to another, without relying on any significance test (Moore & Little, 2024). First, the RI represents the improvement between two, consecutive models’ *LL* relative to the greatest *LL* improvement which occurs when going from 1 profile (variable-centered) to 2 profiles (mixture modeling). Second, the *BF* compares any pair of models the researcher chooses, with support for the model of interest compared to the other model interpreted as moderate with a value of 5.00 and strong with a value of 10.00 or greater. Third, *cmP* calculates a proportion out of 1.00 for the set of models being compared by the researcher with values closer to 1.00 giving more support to a specific model as the best fitting. The *cmP* can be calculated to compare the models within a variance–covariance structure as well as comparing the contender models across the variance–covariance structures.

##### Example data information criteria

Now, we walk through the gestalt decision-making process leading to the determination of the besting fitting models within each variance–covariance structure and then subsequently comparing those models across the variance–covariance structures. Examining the information in Supplemental Table 1 in a gestalt manner, for the diagonal, profile-invariant structure, we have AWE bottoming out at the 5-profile model, and a small change in fit indices from the 5- to 6-profile model, which is also supported by the lower RI and nonsignificant LRT. The 5-profile model also has strong support compared to those models with fewer profiles by the *BF*. The *cmP* supports the 5- and 6-profile models as well. Furthermore, examination of the model results did not help distinguish one model as better from a meaningful, interpretability, or theory perspective.

The diagonal, profile-varying structure, both the AWE and CAIC bottom out at the 3-profile model, which typically suggests a higher profile solution as the best fitting. The BIC bottoms out at the 4-profile solution, giving support to it. We also see the LRT supports the 5-profile solution is not significantly better fitting than the 4-profile solution. Additionally, the 6-profile solution is not significantly better fitting than the 5-profile solution. Examining the *BF* calculated for the 4-profile solution, we see the values are 5 or higher for all models with fewer profiles and with 6 profiles, providing at least moderate support for the 4-profile solution. The 5-profile model *BF* does not support the 5-profile model as a better fitting model than the 4-profile model. Finally, the *cmP* partially supported the 3-, 4-, and 5-profile solutions. Taken together and with the model parameter estimates, the 4-profile and the 6-profile models seemed the top contenders for this structure.

The non-diagonal, profile invariant structure had some fit indices bottom out at the 5-profile model, while the others bottomed out or were lowest at the 7-profile model. The LRTs were nonsignificant for comparing the 5- and 6-profile model pair as well as the 6- and 7-profile model pair. Based on these, the *BF* was calculated for comparing to the 5-profile model and the 7-profile model. The 5-profile model *BF* values strongly supported the 5-profile model compared to models with fewer profiles, but not for profiles with more profiles. The 7-profile model *BF* values strongly supported the 7-profile model compared to all the other models in this structure. Finally, the *cmP* provided minimal support for the 5-profile model, slightly more support for the 6-profile model, and most support for the 7-profile model. Based upon these and the parameter estimates for the models, the 5- and 7-profile models were the top contenders from this structure.

The non-diagonal, profile-varying structure had difficulty with convergence and converging on quality model solutions. Based on the fit indices and the model parameter estimates, the 5-profile model was the best fitting and theoretically interpretable of the models for this structure. Moreover, the *BF* values for the 5-profile model provided strong support for it as the best fitting model.

Finally, the contender models were compared across variance–covariance structures. As with other latent construct models, parsimony is valued. However, this parsimony is not just by number of profiles, but number of overall parameters estimated. This is why for some of the structures, two models were carried forward to this step (a higher and lower profile solution). This step includes more than simply calculating the *cmP* across the variance–covariance structures, but also continuing to examine and compare the parameter estimates and model interpretability of the contender models. The *cmP* supported the diagonal, profile-varying models (4- and 6-profiles). However, examination of the model solutions suggested that the 7-profile, non-diagonal, profile-invariant model was most informative and interpretable. It is important to note that between these two variance–covariance structures, the 7-profile non-diagonal, profile-invariant model constrained the estimates the variances and covariance for ego and task goal orientation across the profiles; while, the diagonal, profile-varying models do not have any covariances estimated, and the variances are uniquely estimated for each profile. Thus, there were more parameter estimates per profile for the diagonal, profile-varying models than for the 7-profile non-diagonal, profile invariant model. Also, most of the variances were not significantly different from each other in the diagonal, profile-varying model; thus, not necessarily adding much useful, interpretable information. To gather further information to help make the final decision regarding the final model to carry forward, the classification quality of all three models was examined.

### Analysis Step 3: investigation of model classification quality

It is important to keep in mind that a well-fitting model does not guarantee high classification quality and for this reason, it is important to assess the classification quality of the models to determine the model that best represents the data and classifies individuals into distinct and meaningful profiles. In this step of the LPA, classification quality statistics, profile separation, and profile homogeneity are examined for the best fitting, contender model(s) (Ferguson et al., 2020; Moore & Little, 2024). As mentioned earlier, after the model is estimated, each individual participant’s probability of profile membership (i.e., posterior probabilities) is calculated based on their indicator responses compared to the profile prototypical responses (Masyn, 2013; Moore & Little, 2024). These individual, profile-specific estimated posterior probabilities are used by the software to calculate profile and whole model classification quality diagnostics, including entropy and average posterior class probabilities, modal class assignment proportion, model estimated class proportions and their confidence intervals (CI). Researchers can also use this information to calculate the odds of correct classification for the profiles (Moore & Little, 2024).

#### Entropy

Entropy is a general, whole model index of overall classification quality for the entire sample, and it is used as an overall indication of how well the individuals are classified into profiles by the model. Ranging between 0 and 1, an Entropy above .80 is considered good (Clark & Muthen, 2009), .70 is acceptable. However, it is not sufficient to determine how well individuals are assigned to profiles, as it is just one, global or gross level, piece of information (Moore & Little, 2024). For the 4-profile and 6-profile, diagonal, profile-varying model entropy was respectively 0.665 and 0.841, while it was 0.829 for the 7-profile, non-diagonal, profile-invariant model.

#### Average posterior class probabilities (AvePP)

AvePPs offers classification quality information about how well individuals are assigned to membership in their most likely (modal) profile (Nylund-Gibson & Choi, 2018). *AvePP* values greater than .70 support the classes are well-separated (Nagin, 2005). Supplemental Table 3 provides the classification quality statistics of the 7-profile, non-diagonal, profile-invariant model. The *AvePP* ranged from .756 to .962, which provides strong support for the classification quality of participants into their modal profile of this model.

#### Modal class assignment proportion (mcaP)

mcaP represents the observed proportion of participants assigned to each profile. The *mcaP* value can be compared to the model estimated profile proportion values (i.e., model estimated parameters) and their 90% or 95% CI. The *mcaP* value falling within the model estimated profile proportion CI provides support for the classification quality of the sample in relation to the model estimated population proportions (Moore & Little, 2024), because the sample proportion falls within the estimated range for the population. All of the 7-profile non-diagonal, profile-invariant model the *mcaP* values were very similar to the model estimated profile proportions, and all fell within the CI.

#### Odds of correct classification (OCC)

OCC represents the odds of participants being assigned to their modal profile by the model solution (i.e., parameter estimates) compared to randomly assigning individuals based on the model estimated profile proportions. Minimal criterion for adequate profile separation and precision in the membership assignment is an OCC value greater than five. The higher the OCC, the greater the evidence supporting the improved classification into participants’ modal profile with the model parameter estimates (Moore & Little, 2024). The OCC values for the 7-profile non-diagonal, profile-invariant model ranged from 18.61 to 626.82, which are all high evidence for the classification quality of this model compared to random assignment.

#### Profile homogeneity and separation

To complete the assessment of the classification quality it is important to explore the profile *homogeneity* and *separation*. All individuals in the sample must be assigned to a profile with other individuals with similar indicator response patterns (i.e., within profile homogeneity) and distinct from those with membership in other profiles (i.e., between profile separation). Therefore, high profile homogeneity and separation indicate good classification quality (Moore & Little, 2024).

When profile variances are freely estimated (i.e., not constrained across profiles), then profile homogeneity can be assessed by calculating for each indicator the ratio of the profile-specific variance divided by the total sample variance for that indicator. Variance proportions less than .60 indicates good homogeneity within the profile compared to the overall sample. Generally, low homogeneity ratio for a single indicator for one profile can be part of what defines the specific profile. It is possible that an indicator with a high homogeneity ratio (i.e., variability more similar to the overall sample) for an indicator does not mean the entire profile or model solution is poor; it may highlight participants’ values on that particular indicator are not as good at distinguishing or separating members of this profiles from the other profiles. In the current, 7-profile non-diagonal, profile-invariant model solution, the variances were constrained across profiles; therefore, the profile homogeneity ratios are not able to provide profile-specific information. However, overall, the profile homogeneity ratios are less than the recommended 0.60 guideline, supporting the profiles are more homogeneous than the overall sample for the task and ego goal orientation variances (Supplemental Table 4).

Profile separation can be assessed by using an adapted version of the pooled formula for Cohen’s *d* (Masyn, 2013; Moore & Little, 2024)*.* A large value (*d* > 2.0) indicates high mean separation (i.e., low distribution overlap) for two profiles on one indicator (Masyn, 2013). Values of *d* < 0.85 indicate more distribution overlap and less separation (Masyn, 2013). It is important to note that every pair of profiles do not need to have high separation on all indicators. To support that two profiles are distinct, they do need to differ on at least one indicator statistically or meaningfully. The right side of Supplemental Table 4 provides the pooled Cohen’s *d* values for each pairwise comparison between the 7 profiles for both the ego and task goal orientation indicators. Overall, the profiles are separate from each other by their mean values for at least one of the indicators. There are only two pairs of profiles (5 vs 6; 5 vs 7) that do not have Cohen’s *d* values exceeding the 2.0 high separation value; however, the absolute values of 1.0 and more extreme still have the majority of the distributions of the profile indicators distinct (i.e., not overlapping). Additionally, when examining the 95%CI for the mean indicator values, there are significant differences between the profile means (See Supplemental Table 4).

Finally, separation can also be described by the distinctiveness of the variances and covariances of the indicators when they are estimated uniquely for the profiles, as the variances and covariances are then part of the measurement model of the profiles. How different the variance–covariance parameter estimates are between the profiles can be derived by using the 95% CI for the profile-specific variance and covariance point estimates for the indicators. No overlap of 95% CIs for a variance or covariance indicates the two profiles’ separation can be described by that parameter. This separation can also be shown using the profile-specific standard deviations and correlations estimated by the model (Masyn, 2013).

#### Profile plots and other graphical representations

Profiles can be visually represented by a profile plot—a line graph of the means of the indicators for each profile—to clearly see the individual profiles and quantitative differences. Including the 95% CIs in the profile plots makes it easier to see whether two profiles overlap. Lack of overlap suggests the mean values for that indicator variable are distinct between those profiles, showing separation support. Given the number of profiles in the example, the profile plot was instead designed to have a line represent the task and ego goal orientation values respectively by profile (Supplemental Fig. 2). Alternatively, LPA profiles can be visually represented with a bar graph of the standardized indicator responses for each profile, clearly showing how the profiles compare to each other relative to the sample mean for each indicator. When there are freely estimated covariances as part of the measurement model for the profiles, this can be visually represented with bivariate scatterplots of individuals’ responses that include the estimated class-specific correlation trend lines and ellipses around each class centroid to represent the class-specific variability of the responses about the two indicators (Masyn, 2013).

#### Profile naming

All the above-mentioned information can inform the profile naming process. When naming profiles, it is important to keep in mind the response range for the indicator variables and the interpretation of the response scale, and potentially the observed differences in indicator values. For example, Profile 1 (Supplemental Table 4) has the lowest task orientation of all the profiles; however, to describe the profile members as not holding a task goal orientation is inaccurate, since the mean value of 4.01 is the mid-point of the response options. Profile 1 members average 2.88 on their ego orientation which is disagreeing with holding an ego orientation. Thus, we could describe Profile 1 members as not having strongly developed their goal orientations; they do not define success in PE by comparison with others, and only minimally by their own self-referenced improvement and learning. Overall, this profile is a rather neutral goal orientation profile. For comparison, Profile 3 members slightly disagree with an ego orientation, while slightly agreeing with holding a task orientation. Thus, these profile members define success in PE with a self-referenced approach, just not strongly. Finally, members of Profile 7 could be described as having strongly adopted a task goal orientation, and not adopting an ego goal orientation. Noteworthily, when naming profiles, it is necessary to carefully avoid suggesting the assigned name is correct, being judgmental of members, or proving the existence of the profile because it is named (Kline, 2011; Masyn, 2013).

### Covariate analysis

Once the final model is identified the covariates can be included (Nylund-Gibson & Masyn, 2016; Wang & Wang, 2020), including covariates should be done if (a) there are profiles worth interpreting further and (b) there are theoretical reasons leading to the exploration of the relationships between the covariates and the profiles (Ferguson et al., 2020). Exploring the relationships between the profiles and the covariates can offer validity information for the latent profiles (Masyn, 2017; Moore & Little, 2024; Spurk et al., 2020).

Covariates can be included as either predictors or outcomes of profile membership. Predictors of profile membership can be interpreted as the proportion of profile members represented for different covariate values or as the odds of profile membership relative to the value of the covariate. Instead, outcomes of profile membership can be presented as means based upon profile membership and can be tested for significant differences. If after all of the prior LPA steps of enumeration and examining classification quality, homogeneity and separation, there is still more than one contender model the researcher is trying to decide between, those models can be carried forward to include covariates to have validity evidence to help with the final decision process. When this is the case, as with all analysis steps, it is important to be transparent in reporting how the validity information helped inform the final decision on the best fitting model.

It is important to understand the challenges that arise from adding covariates into finite mixture models and why the covariates are added separately from the profile enumeration process. The reasons for this differ for predictors and outcomes. When a predictor is added to the model, the relationships between the predictor and the indicators is indirectly explained through the profile construct. When this indirect relationship fully explains the predictor-indicator relationships, then the profiles remain stable. However, when the indirect relationship does not fully explain the predictor-indicator relationships, then the profiles shift in proportions and potentially parameter estimates as well. This shifting is due to differential item functioning (DIF) of one or more indicators in relation to the predictor (Masyn, 2017). When there is minor DIF, the effect on the profiles is also minimal. However, with large or significant DIF, the carefully enumerated, meaningful profiles shift significantly. This DIF can be tested for and then directly modeled to stabilize the profiles and have this result information be part of the interpretation (see Masyn, 2017; Nylund-Gibson et al., 2019). Alternatively, to maintain the enumerated measurement structure, approaches have been developed to separate the enumeration and covariate analyses (described below). When outcomes are added to the model, the outcomes are treated the same by the software as the indicators of the profiles; therefore, it is important to have enumerated and stabilized the profiles so that the carefully enumerated profiles are maintained when the outcomes are added in for validity and interpretation purposes.

While we will focus on the two most established approaches for adding covariates to finite mixture models while maintaining the enumerated profiles are the ML 3-step (or VAM) approach (Bolck et al., 2004; Vermunt, 2010) and the BCH approach (Bakk & Vermunt, 2015), there are others, such as the two-step approach (Bakk & Kuha, 2021). Both the three-step and BCH approaches use the participants’ modal profile assignment and incorporate the uncertainty of that assignment. The ML 3-step approach incorporates the assignment uncertainty at the profile-level using the average posterior probabilities in logit metric. The BCH approach incorporates the assignment uncertainty at the individual level with BCH weights uniquely calculated for each person by the software. These two approaches are important advances on the hard assignment approach used previously. With hard assignment, participants were assigned to their modal profile, which was then treated as a known grouping variable either as an outcome of logistic regressions to predict profile membership or as the independent variable of an ANOVA to determine mean differences by profile membership. The weakness of this approach in weighting all participants assigned to a profile equally without including uncertainty is that a participant modally assigned to this profile with a posterior probability of .30 is given equal weight as a participant modally assigned to this profile with a posterior probability of .99; this reduces the power to detect profile differences like using a median split to create groups (Field, 2018).

These approaches can be used to test covariate relationships with profiles in M*plus* by either writing syntax (i.e., manually) or using automatic covariate inclusion syntax with either the command (R3STEP) after predictors or (BCH) after outcomes listed in the AUXILIARY command. The automatic VAM (i.e., R3STEP command) and BCH approaches in M*plus* currently only show results for covariates in the output when the inclusion of the covariates does not result in much profile shifting. If there are no results in the output related to the covariates then the inclusion of the covariates resulted in profile shifting that exceeded M*plus*’s acceptability criterion. The other limitation of these automated approaches is that only predictors or outcomes can be examined in relation to the profiles. However, manually specifying covariate inclusion with syntax using either VAM or BCH approaches allows for the inclusion of any combination of predictors, outcomes, or moderation of other constructs’ relationships by the profiles (Masyn, 2013; Moore & Little, 2024). However, it is also important to confirm the profiles remain the same once the covariates are included in the model (Fergusson et al., 2020), as such fluctuations may indicate a misfit due to the relationship between the covariate and one or more indicators being greater than what is represented in the model (cf. Masyn, 2017). Moore and Little’s (2024) synthesis of simulation research on which of these two approaches to use when bringing in covariates highlighted the following trends: a) VAM performs better with low sample size, class separation, entropy, or when there is large missing on the covariates; and b) BCH performs better with large sample size, strong entropy, or low missing on covariates. However, if the selected approach results in profile shifting, then it is appropriate to attempt the other approach for improved stability.

#### Covariates and missing data

It is noteworthy most software listwise deletes participants with missing data on profile predictor covariates. However, this is not recommended as it leads to biased parameter estimates and changes in the number of participants in some profiles. There are options to address this. One approach is to model single-variable covariates as single-indicator latent constructs, which then have any missingness handled by the ML estimator. Another option is to impute the predictors after the BCH weights have been added (Nylund-Gibson & Masyn, 2016; Nylund-Gibson et al., 2019).

##### Including covariates with the data example

The covariates were added using the VAM method given the mid-range for the sample size and the entropy value. First, gender was added (Supplemental Syntax Appendix 10) and with an omnibus test to determine if gender identity significantly predicted any profile membership. The profile integrity was maintained, and gender identity significantly predicted profile membership (*p* < .001). Probabilities of profile membership by gender identity were calculated and graphed (Supplemental Fig. 3) to help interpret and visualize the significant differences. Specifically, women compared to men were significantly more likely to be in a) Profile 7 compared to Profiles 1, 2, 3, and 6; and b) Profile 5 compared to Profiles 2 and 3.

The outcomes of enjoyment and boredom were added to the model next and regressed on the gender identity predictor to account for any gender differences. Figure 4 presents the profile means of enjoyment and boredom adjusted for gender identity. Pairwise comparisons conducted within M*plus* (Supplemental Syntax Appendix 11) supported the following significant (*p* < .001) differences: a) Profile 2 members reported significantly higher PE enjoyment than members of Profiles 1, 3, 5, 6, and 7; Profile 1 members reported significantly lower PE enjoyment than members of Profiles 2, 4, 5, and 7; and c) Profile 7 members also reported significantly higher PE enjoyment than members of Profiles 3 and 5. Additionally, boredom in PE reported by members of Profile 1 was significantly (*p* < .001) higher than that reported by members of any other profile. These results, in addition to the significant profile membership prediction by gender identity, provided validity support for the 7-profile non-diagonal, profile-invariant solution.

## LPA vs variable-centered regression model

Whether to conduct person-centered vs variable-centered analyses is not necessarily an either-or dichotomy (Masyn, 2013); rather it is dependent on researchers selecting the more appropriate analysis from their analytical toolbox to address their research question. With our example data, the variable-centered regression simply told us one’s task goal orientation significantly predicted enjoyment positively and boredom negatively; while ego goal orientation did not significantly predict either outcome. On the other hand, by using the person-centered LPA, we saw those without a definition of success (Profile 1) had significantly greater boredom than everyone else and less enjoyment than most. The two profiles with the highest enjoyment and lowest boredom values had similarly high task orientation adoption, however, Profile 2 members also endorsed an ego orientation, whereas Profile 7 members did not endorse an ego orientation. In this example, the LPA result helped explain why ego orientation rarely shows significant bivariate relationships with enjoyment or boredom. Thus, examining the goal orientation-based profiles gave more nuanced, informative, and real-world representations of how people define success in their fitness classes, and how those definitions together influence their enjoyment and boredom experiences. This example illustrates why it is important to use the analytic method that best represents the theory, what is occurring within people (adopting independent levels of task and ego goal orientations), and the researcher’s question.

## Conclusion

This article introduced the general purpose and concept of finite mixture models. Then, given the continuous nature of most behavioral medicine variables, we walked through the analysis steps conceptually and concretely for conducting LPAs with an exercise psychology data example. This started with development of research questions and hypotheses through profile enumeration and ended with inclusion of covariates and interpreting those results. The supplemental material includes the tables and figures used to represent the results of these steps, as well as examples of syntax for each step, and the relevant dataset. The advancement in computational power now facilitates researchers conducting LPAs, and finite mixture models in general, to answer previously unexamined research questions. As illustrated by the exercise psychology example, finite mixture models can be theoretically informed just like variable-centered analyses. However, LPAs allow for testing aspects of theories that do not fit a variable-centered analysis framework, such as how individuals’ simultaneous ego and task orientation levels relate to their motivational responses in group fitness classes. There are aspects of finite mixture models that are continuing to develop such as other ways to include covariates (McLarnon & O’Neill, 2018), fit indices (Grimm et al., 2021), and how to analyze imputed data in a finite mixture model framework (Waldman, 2020), so it is important to use current best practice recommendations when conducting these analyses. This article covered current best practices that have been tested and accepted by quantitative methodologists.

## Data Availability

The data, analysis syntax, tables, and figures for the example are accessible via the OSF link: https://tinyurl.com/ExampleLPA.

## Footnotes

1 The first author’s IRB declared the overall study except (See Lucas and Moore, 2019 for details). All participants assented to participate after reading an information sheet.

2 We encourage researchers to confirm the specificities of their chosen software regarding which distributions it can handle in combination with finite mixture models if this need is anticipated.

3 For example, M*plus* supports 10,000 models with random starts beginning in a model attempt and taking 5,000 through the convergence process.
